# Multiple Small Axillary Masses With Haloes on Ultrasound Mimicking Lymph Node Metastases in Breast Cancer: A Case Report

**DOI:** 10.7759/cureus.100777

**Published:** 2026-01-04

**Authors:** Takeshi Suto, Shoji Oura

**Affiliations:** 1 Department of Surgery, Kishiwada Tokushukai Hospital, Kishiwada, JPN

**Keywords:** axillary recurrence, axillary small nodules, breast cancer, haloes, thick flap

## Abstract

A 66-year-old woman had presented with various oncological events, such as in-breast recurrence, local recurrence, contralateral axillary lymph node recurrence, left ductal carcinoma in situ (DCIS) of the breast, and primary unknown metastatic neuroendocrine tumor of the liver, after neoadjuvant chemotherapy followed by breast-conserving therapy with sentinel node biopsy (SNB) for right breast cancer, i.e., invasive micro papillary carcinoma.

Attending surgeons had managed these events with salvage mastectomy and re-SNB, wide resection, axillary lymph node dissection, nipple-sparing mastectomy in a thick flap manner, and partial hepatectomy, respectively. Follow-up positron emission tomography/computed tomography further showed multiple fluorodeoxyglucose uptakes in the left axilla. Ultrasound showed multiple small lesions with internal low echoes, obscured margins, and haloes. Core needle biopsy pathologically showed atypical cells growing in a trabecular and solid fashion without any lymph node structures. Under the tentative diagnosis of in-breast recurrence of the left DCIS in the preserved thick flap, the patient underwent resection of the multiple axillary lesions. All nine lesions showed estrogen receptor-positive and human epidermal growth factor type 2 receptor-negative cancer cells with a very low Ki-67 labelling index of 2%, highly resembling the pathological findings of the left DCIS.

These images and pathological findings suggest that multiple small axillary masses with haloes on ultrasound are not lymph node metastases of breast cancer. Breast specialists, therefore, should develop therapeutic strategies based on the idea that multiple small axillary nodules with haloes on ultrasound have a high probability of in-breast recurrence.

## Introduction

Breast surgeons have had more opportunities to diagnose and treat axillary recurrence after the clinical introduction of sentinel node biopsy (SNB) [1]. The vast majority of axillary recurrences develop due to inappropriate surgical procedures of SNB and often can be cured with salvage axillary dissection, like salvage mastectomy for in-breast recurrence after breast-conserving therapy. Breast surgeons, therefore, should detect axillary recurrence at its earliest phase for better clinical outcomes.

It is well known that when breast cancer cells infiltrate into the subcutaneous fat, band-like high echoes, known as haloes, are observed just above the breast cancer. It is also known that high echoes called haloes are generated when ultrasound waves are backscattered by breast cancer cells present in the fat tissue [2]. Thus, accompanying haloes generally observed around breast cancer naturally make diagnostic physicians judge the lesion to be invasive breast cancer with fat invasion.

We experienced a right breast cancer patient who later developed multiple recurrent and non-recurrent oncological events such as in-breast recurrence, local recurrence, contralateral axillary lymph node recurrence, left ductal carcinoma in situ of the breast, primary unknown metastatic neuroendocrine tumor of the liver, and multiple small nodules with haloes in the left axilla, and herein report no correlation between multiple small tumors with haloes on ultrasound and lymph node recurrence in the axilla through the pathological findings and halo formation mechanisms on ultrasound [3].

## Case presentation

**Figure 1:**
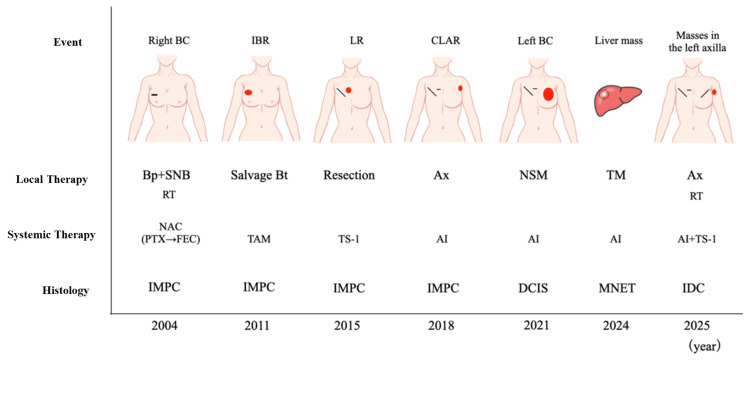
Time course of oncological events, histology and treatment. Events in this case included primary breast cancer, in-breast recurrence, local recurrence, contralateral axillary lymph node recurrence, contralateral breast cancer, liver metastasis from a neuroendocrine tumor of unknown primary site, and contralateral axillary recurrence. BC: breast cancer, IBR: in-breast recurrence,  LR: local recurrence, CLAR: contralateral axillary lymph node recurrence, Bp: partial mastectomy, SNB: sentinel node biopsy, Bt: mastectomy, Ax: axillary dissection, NSM: nipple sparing mastectomy, TM: tumorectomy, RT: radiotherapy, NAC: neoadjuvant chemotherapy, PTX: paclitaxel, FEC: fluorouracil, epirubicin, cyclophosphamide, TAM: tamoxifen, TS-1: tegafur, gimeracil, oteracil potassium, AI: aromatase inhibitor, IMPC: invasive micro papillary carcinoma, DCIS: ductal carcinoma in situ, MNET: metastatic neuroendocrine tumor, IDC: invasive ductal carcinoma.

A 66-year-old woman had undergone breast-conserving therapy and SNB after neoadjuvant chemotherapy usingth weekly paclitaxel chemotherapy followed by anthracycline-containing chemotherapy. The patient developed in-breast recurrence seven years later and therefore underwent salvage mastectomy and re-SNB. The patient further developed local recurrence and contralateral axillary lymph node recurrence in another four and seven years, respectively, making attending surgeons treat each recurrence with surgery. We got postoperative pathological reports that all these primary and recurrent foci were invasive micro papillary carcinomas (Figure 1).

**Figure 2:**
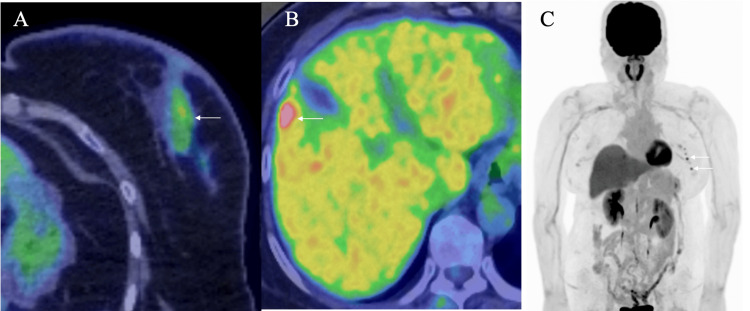
Positron emission tomography/computed tomography (PET/CT) findings. A. PET/CT showed a slightly elevated maximal standardized uptake value of 4.23 (arrow)  in the left breast; B. PET/CT showed that the liver lesion had avid fuorodeoxyglucose (FDG) uptake (arrow) in the liver segment 5, i.e., a maximal standard uptake value of 14.3; C. Coronal view PET/CT showed avid FDG uptake in multiple small lesions  (arrow) in the axilla, i.e., a maximal standard uptake value of 5.67.

Thereafter, follow-up positron emission tomography/computed tomography (PET/CT) clarified the fluorodeoxyglucose (FDG) uptake in the left breast without any symptoms around the left axilla (Figure 2A, 2B).

**Figure 3:**
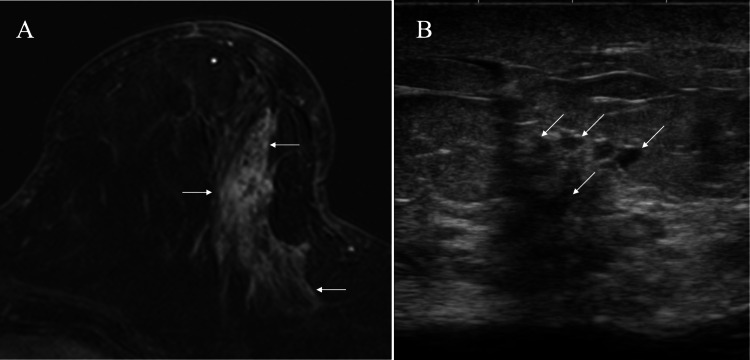
Magnetic resonance imaging (MRI) and ultrasound findings of the left breast cancer. A. MRI showed enhancement extending widely from the subnipple areas toward the left axilla (arrows); B. Ultrasound showed multiple non-mass forming lesions with internal low echoes (arrows).

Ultrasound and magnetic resonance imaging showed non-mass-forming lesions spreading widely in the left breast (Figure 3).

**Figure 4:**
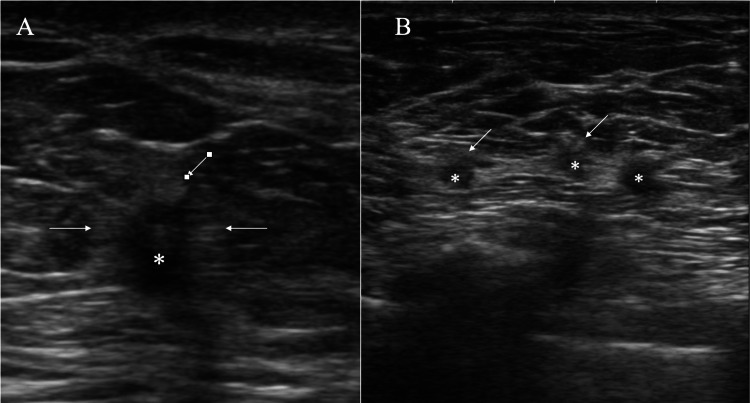
Ultrasound findings of the axillary lesions. A. Ultrasound showed the largest lesion (asterisk), less than 1cm in size, with predominant internal low echoes and haloes (arrows) surrounding the mass; B. Ultrasound showed multiple small lesions(asterisks) with haloes (arrows) lining in line.

Core needle biopsy pathologically showed low-grade ductal carcinoma in situ (DCIS) of the left breast, which was treated with nipple-sparing mastectomy in a thick flap manner. Immunostaining of the DCIS showed estrogen and progesterone receptor (RR and PgR) positivities (Allred scores 8 and 5, respectively), human epidermal growth factor receptor type 2 (HER2) negativity, and a low Ki-67 labelling index of 1.5% [4]. In another three years, PET/CT showed small FDG uptake in the liver (Figure 2B). We, therefore, resected the hepatic lesion both for cure and pathological diagnosis of the hepatic mass, and got the diagnosis of metastatic neuroendocrine tumor of unknown primary. We, therefore, decided to follow up further because breast cancer-related events continued even though 20 years had passed since the initial breast cancer treatment. Another PET/CT further showed multiple avid FDG accumulations in the left axilla one year after the liver operation (Figures 1, 2C). Ultrasound showed multiple small nodules with indistinct margins, low internal echoes, slightly attenuated posterior echoes, and haloes around the masses (Figure 4).

**Figure 5:**
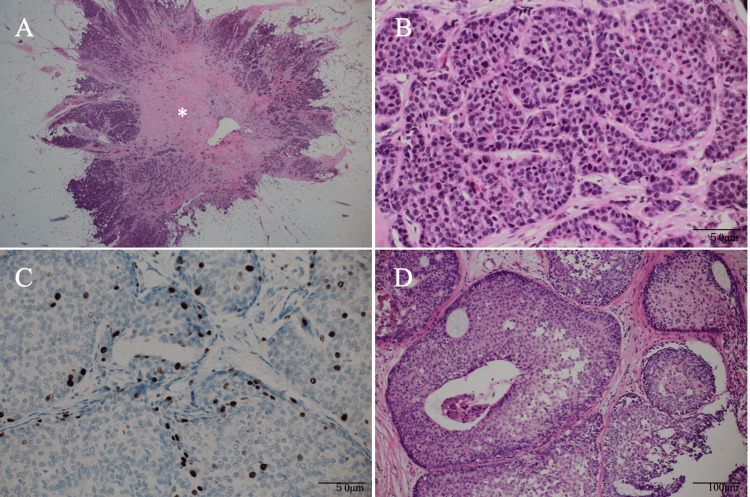
Pathological findings. A. Low magnified view showed an irregular mass which had abundant fibrous components (asterisk) in its center and no lymph node structures; B. Magnified view showed atypical cells growing in trabecular and solid fashions; C. Immunostaining showed a very low Ki-67 labelling index of 2%; D. Magnified view of the left ductal carcinoma in situ showed similar pathological findings to those observed in the axillary lesions.

Pathological study of the core needle biopsy specimen of the axillary mass showed small, round atypical cells growing in solid and sheet-like fashions with ER positivity. The patient had been well without any symptoms despite many prior treatments. Therefore, under the tentative diagnosis of in-breast recurrence developed from the minute residual mammary glands in the preserved thick subcutaneous fat, we resected all small nodules in the axilla. All nine lesions lacked lymph node structures within them and had invasive cancer cells growing in trabecular and solid fashions, highly resembling the pathological findings of the left DCIS. Immunostaining showed ER positivity (Allred score 7), PgR negativity, HER2 negativity, and a very low Ki-67 labelling index of 2% (Figure 5).

The patient is scheduled to receive adjuvant radiotherapy to the left breast and is going to make the shared decision with us about the systemic therapy after finishing the radiotherapy.

## Discussion

Given the prior surgeries both to the contralateral lymph node recurrence in the left axilla and nipple sparing mastectomy in a thick flap fashion for DCIS in the left breast, diagnostic physicians can speculate two recurrence mechanisms against the multiple small axillary lesions such as re-recurrence of the right invasive micropapillary carcinoma to the contra-lateral axilla and growth of the residual DCIS in the minute mammary gland present around the axilla [5]. It is natural for us to judge that these axillary lesions should be in-breast recurrence after four years of disease-free interval due to the similar pathological findings to those of the left DCIS, lack of pathological lymph node structures, ER positivity, HER2 negativity, and very low Ki-67 labeling indices between the recurrent foci and the DCIS in the left breast [6].

Ultrasound showed haloes just around all small axillary lesions in this case. Cancer cell infiltration, if present, generates haloes on ultrasound not only in the subcutaneous fat just around breast cancer, but also in the axillary fat by ultrasound wave backscattering [7]. Therefore, if the haloes observed in this case had been caused by metastatic lymph nodes, cancer cells would have spread beyond the lymph node capsule despite the absence of lymph node enlargement, leading to the contradiction between the image and pathological findings.

This patient had presented very complicated oncological events, such as in-breast recurrence, local recurrence, rare contralateral axillary lymph node metastasis, contralateral DCIS, and the primary unknown metastatic neuroendocrine tumor to the liver [3]. In addition, it is well known that core needle biopsy specimens of the lymph node metastasis sometimes have no lymph node structures. Some physicians, therefore, would have treated this patient not with surgery but with endocrine therapy under the non-curative intent if they had assessed these axillary lesions to be metastatic lymph nodes from the right breast cancer and had taken the presence of a still undetected primary neuroendocrine tumor into consideration. However, if these axillary lesions had a pathogenesis similar to in-breast recurrence after breast-conserving therapy, they may be cured by surgical treatment [8].

Diagnostic physicians generally make diagnoses of various types of recurrences based on their own experience of such clinical cases, but may sometimes correctly evaluate the breast cancer recurrence when having a prior complex clinical course. In fact, we struggled with the clinical question of whether to perform hormone therapy or primary surgical treatment because the axillary recurrence in this case was ER-positive. However, when evaluating the low Ki-67 labeling index and ultrasound findings together, it is possible for many physicians to determine that many small lymph node metastases extremely rarely have extracapsular invasion. This case clearly demonstrates that by understanding the mechanisms of halo formation on ultrasound from the perspective of acoustic impedance, more appropriate treatment can be selected for breast cancer recurrence [9].

## Conclusions

We experienced a case of multiple small recurrent foci in the axilla with haloes on ultrasound after many oncological events. We concluded from pathological examination that this recurrence was caused by breast cancer surgery using thick flaps. Breast surgeons should be aware that breast cancer surgery using thick flaps may result in breast recurrence, like this case, and must treat such recurrence with surgical intervention. In addition, breast specialists should note that multiple small axillary masses with haloes on ultrasound are not lymph node metastases of breast cancer.
